# Modulation of Milk Source Differences on Immunity, Nutritional Physiology and Intestinal Microbiota in Neonatal Piglets

**DOI:** 10.3390/ani15213104

**Published:** 2025-10-25

**Authors:** Junhong Liu, Miaomiao Bai, Shanshan Wang, Yihui Zhang, Changfeng Peng, Yirui Shao, Xia Xiong, Yueyao Xing, Hongnan Liu

**Affiliations:** 1 Hunan Provincial Key Laboratory of Animal Nutritional Physiology and Metabolic Process, National Engineering Laboratory for Pollution Control and Waste Utilization in Livestock and Poultry Production, Institute of Subtropical Agriculture, Chinese Academy of Sciences, Changsha 410125, China; 2 College of Animal Science and Technology, Northeast Agricultural University, Harbin 150030, China; 3 Ausnutria Dairy (China) Co., Ltd., No. 2, Wangwang East Road, Wangcheng District, Changsha 410219, China; 4 College of Animal Science and Technology, Hunan Agricultural University, Changsha 410128, China

**Keywords:** goat milk formula powder, neonatal piglets, growth performance, nutritional physiology, intestinal microbiota

## Abstract

Milk sources directly influence digestion, absorption, and overall nutrient utilization during early infant nutrition. Goat milk features a nutritional composition and digestive properties that are more similar to human breast milk. This study aimed to investigate the effects of different milk sources on the immunity, amino acid and fatty acid metabolism, and intestinal microbiota in neonatal piglets. Sixteen 7-day-old suckling piglets were randomly allocated into two groups (eight replicates/group, one piglet/replicate) and fed with standard formula milk powder (CON) and goat milk formula powder (GMF). The formal experiment lasted for 14 days. Results showed that compared with the CON group, the GMF group showed a significant increase (*p* < 0.05) in the final weight, the serum levels of immunoglobulin A (IgA), IgG, IgM and C-reactive protein (CRP4), and intestinal trypsin content. Additionally, the GMF group had higher (*p* < 0.05) serum essential and non-essential amino acid and fatty acid levels, and had trends toward upregulation (0.05 < *p* < 0.1) in hepatic mRNA expression of spermine N1-acetyltransferase 1 (*SAT1*), duodenal peptide transporter 1 (*PePT1*), and jejunal cationic amino acid transporter 1 (*CAT1*). Microbiome sequencing revealed that GMF enhanced intestinal microbial richness and diversity and increased concentrations of acetic and propionic acids (*p* < 0.05). In conclusion, GMF suggests a potential improvement in the growth performance by enhancing immunity, amino acid and fatty acid metabolism and optimizing intestinal microbiota composition in neonatal piglets. These findings further support the favorable nutritional properties and tolerability of GMF in early-life nutrition.

## Simple Summary

Differences in milk sources exert a considerable influence on the early digestive and absorptive processes, allergy susceptibility, and nutritional utilization in neonatal piglets. The nutritional profile and digestive properties of goat milk powder are more close to those of breast milk, facilitating easier digestion and absorption in infants. Utilizing neonatal piglets as an animal model, the present study aims to investigate the effects of different milk sources on immune function, amino acid and fatty acid metabolism, and intestinal microbiota. Findings indicate that goat milk formula powder showed promising trends in neonatal piglets’ growth performance by augmenting immune responses, promoting amino acid metabolism, and modulating the intestinal microbiota, thereby demonstrating its superiority over cow milk formula powder.

## 1. Introduction

Human milk is the optimal infant nutrition [1], yet the global exclusive breastfeeding rate is only 38%, making infant formula a necessary alternative [2,3]. Milk serves as a primary ingredient in most formulas [4], yet cow’s milk allergy affects 2–3% of infants under one year old, primarily triggered by whey proteins and caseins [5]. Consequently, alternative milk sources like donkey, mare, and camel milk have been investigated due to their potentially reduced allergenicity [6,7]. Comparative studies indicate that immunological cross-reactivity with cow’s milk generally follows the order: cow milk > goat milk > camel milk [8,9]. Evidence demonstrates that goat milk is a promising alternative source of oligosaccharides to bovine milk for use in infant formula [10], and goat milk powder is expected to become a better infant formula than cow milk powder.

Goat milk offers several distinct advantages. Notably, goat milk forms a looser coagula under acidic conditions, facilitating protease penetration and casein breakdown [11]. Goat milk exhibits distinct advantages over cow milk, such as smaller milk fat globules and more short/medium-chain fatty acids, enhancing protein digestibility [12,13,14]. Xu et al. [15] demonstrated that GMF improves early growth and immune function in rats after weaning. Under simulated infant gastric conditions, goat milk formula powder demonstrates physicochemical and proteolytic similarities to cow milk formula powder [16]. These properties position goat milk formula powder as a safer alternative for cow’s milk allergy management, alleviating gastrointestinal symptoms and parental concerns [17]. Piglets are an adaptable and robust model for pediatric nutrition and metabolism research, with demonstrated physiological parallels to human infants in nutritional physiology, intestinal development, and brain development [18]. Based on the documented beneficial properties of goat milk and the physiological relevance of the piglet model, we hypothesize that a GMF will enhance immunity, improve amino acid and fatty acid metabolism, and modulate intestinal microbiota in neonatal piglets. This study aims to optimize infant formula design and inform clinical applications.

## 2. Materials and Methods

### 2.1. Experimental Design and Diets

The experiment was conducted at the Institute of Subtropical Agriculture, Chinese Academy of Sciences. Piglets were humanely euthanized via intravenous pentobarbital sodium administration under deep anesthesia to ensure painless death. This experiment followed guidelines for animal research approved by the Animal Welfare Committee of the Institute of Subtropical Agriculture, Chinese Academy of Sciences (Changsha, ISA20240017). Sixteen healthy 7-day-old male piglets with similar body weights (BW = 2.23 ± 0.40 kg) were randomly divided into two groups. They were orally administered standard formula milk powder (CON) and goat milk formula powder (GMF) via feeding bottles, respectively. Each group consisted of 8 replicates, with 1 piglet per replicate, and the piglets were individually housed in infant incubators that maintained a constant temperature (30–32 °C). The pre-feeding period lasted for 3 days, followed by a formal experimental period of 14 days. The feeding procedure followed the protocol established by Bai [19]. The daily feed allowance was adjusted each day based on the piglet’s body weight, calculated at a rate of 42 g DM/kg. Each pig had access to milk every 2 h from 07:30 to 24:00 in each group. The occurrence of bloating and milk spitting in piglets was observed and recorded. The formula milk powder used in this experiment was provided by Ausnutria Dairy Co., Ltd. (Changsha, China), under the brand name Kabrita. The selected piglets were obtained from a commercial pig farm. The nutritional composition of different infant formulas is presented in Table 1.

### 2.2. Sample Collection

Before trial completion, piglets were fasted for 12 h, anesthetized, and then euthanized. Blood samples were collected from the anterior vena cava using sterile 10 mL vacuum tubes without anticoagulant. After clotting for 2 h at room temperature, the samples were centrifuged at 3000× *g* for 10 min at 4 °C using a refrigerated centrifuge (Xiangyi Centrifuge Instrument Co., Ltd., Changsha, China). The resulting serum was carefully separated and stored at −80 °C in a refrigerator (Haier DW86L578J, China). Tissue samples and intestinal content were immediately snap-frozen in liquid nitrogen and stored at −80 °C for subsequent use within six months.

### 2.3. Determination of Growth Performance Indexes

Piglets were weighed daily before feeding. Based on initial and final body weight, average daily gain (ADG) was calculated. The milk consumption of piglets was recorded every day, and the average daily feed intake (ADFI) and feed to weight FCR (Feed Conversion Ratio) = ADFI/ADG were calculated.

### 2.4. Determination of Serum Biochemical Indexes

The Beckman CX4 automatic blood biochemistry analyzer [20] (Beckman Coulter, Inc., USA) was used to detect serum total protein (TP), blood urea nitrogen (BUN), albumin (ALB), alanine aminotransferase (ALT), glucose (GLU), aspartate aminotransferase (AST), alkaline phosphatase (ALP), CRP4, total cholesterol (CHOL), cholinesterase (CHE2), low-density lipoprotein (LDL-C), triglycerides (TG), Hepatic Lipase (LIPC), high-density lipoprotein (HDL-C). The kits were purchased from Beijing Leadman Biochemistry (China).

IgA, IgG, and IgM were measured using ELISA kits, and all experimental procedures were strictly performed according to the manufacturer’s instructions (Nanjing, China). Complement C3 (C3) and Complement C4 (C4) were determined using kits purchased from Beijing Leadman Biochemical Technology Co., Ltd. (Beijing, China), following the provided protocols.

### 2.5. Measurement of Serum Free Amino Acids

The process of measurement was as follows: Draw 1 mL of serum into a centrifuge tube, and then add 1 mL of 8% sulfosalicylic acid. After thorough mixing, place the tube in a 4 °C refrigerator for 15 min of standing. Subsequently, centrifuge the mixture at 10,000 rpm for 10 min. Carefully aspirate the supernatant, filter it through a 0.22 μm membrane, and transfer the filtered solution into a clean container for storage. For amino acid analysis, use an amino acid analyzer (Hitachi L8900, Tokyo, Japan) with the following parameters: injection volume of 20 μL, analysis cycle of 150 min, and column equilibrium time of 35 min. For detection wavelength, all amino acids were measured at 570 nm, except for proline (Pro), which was quantified at 440 nm [21].

### 2.6. Assessment of mRNA Expression Levels of Liver, and Intestinal Related Genes

Total RNA was extracted from approximately 0.2 g of sample using the TRIzol kit (Accurate Biology, Changsha, China) according to the manufacturer’s instructions. RNA purity and concentration were measured with an ultra-microvolume spectrophotometer (IMPLEN, Germany). The RNA was then diluted to a uniform concentration based on the measurements and reverse-transcribed into cDNA in a 20 μL reaction system using the Prime Script TMRT Master Mix kit (Accurate Biology, Changsha, China). Finally, quantitative real-time PCR (qPCR) was performed using the TB Green Premix Ex Taq reagent kit (Accurate Biology, Changsha, China) on a LightCycler 480II system (Roche, Basel, Switzerland) [22]. Gene and primer sequences are presented in Table 2.

### 2.7. Determination of Long-Chain Fatty Acids in Serum

A 1–10 mL serum sample was placed in a centrifuge tube, mixed with 2 mL of 5% acetyl chloride/methanol solution (*v*/*v* = 1:19), vortexed, sealed, and incubated overnight at 50 °C in a water bath shaker (Zhchenc ZWF-110X30, Zhengzhou, China). After incubation, 1 mL of n-hexane was added and the mixture was vigorously shaken and then centrifuged at 3000 rpm for 5 min. The supernatant was transferred to a new tube, dried with anhydrous sodium sulfate, and then aspirated, filtered (0.22 μm), and analyzed by gas chromatography (Agilent Technologies Inc., Palo Alto, CA, USA).

### 2.8. Determination of Intestinal Digestive Enzymes

The contents of digestive enzymes including α-amylase, chymotrypsin, lactase, trypsin, and lipase in jejunal mucosal samples of piglets were measured using ELISA kits, with all experimental procedures strictly following the manufacturer’s protocols (Nanjing Jiancheng Bioengineering Institute, Nanjing, China) [23].

### 2.9. Gut Microbial Community Analysis

Genomic DNA was extracted from colonic content samples using a fecal genomic DNA extraction kit, with the DNA purity and concentration assessed by agarose gel electrophoresis. PCR amplification was conducted using a high-fidelity PCR enzyme system (GC Buffer, New England Biolabs) with uniquely barcoded primers. The amplification products were verified by 2% agarose gel electrophoresis. Sequencing libraries were constructed using the Ion Plus Fragment Library Kit (48 reactions, Beijing Novogene Technology Co., Ltd., Beijing, China) [22]. After quantification with Qubit and quality control verification, sequencing was performed on the Ion S5 XL system (Thermo Fisher Scientific, Waltham, MA, USA). The 16S rRNA gene sequences have been deposited in the NCBI Sequence Read Archive under accession number PRJNA1255375.

### 2.10. Colonic Short-Chain Fatty Acids Content Detection

Fresh colonic contents (1 g) were homogenized with 5 mL ultrapure water by vortexing for 30 min, followed by 4 °C overnight incubation. After centrifugation (10,000 rpm, 10 min), the supernatant was collected and the pellet was re-extracted with 4 mL ultrapure water for 30 min. The supernatant was centrifuged at 12,000 rpm for 15 min and transferred to a new supernatant. The mixture was then added to a centrifuge tube at a ratio of *v*:*v* = 9:1 (900 μL of new supernatant + 100 μL of 25% phosphoric acid). After mixing the liquid, it was allowed to stand at room temperature for 3 h. It was then filtered through a 45 μm micropore into an upper sample bottle and tested by gas chromatography–mass spectrometry (Agilent 7890A, Agilent Technologies Inc., Palo Alto, CA, USA). Methods were carried out as described by [22].

### 2.11. Statistical Analysis

All the results were expressed as means ± SEM. This experiment utilized GraphPad Prism 10.4 (GraphPad Software, Inc., Boston, MA, USA) for data processing. After assessing normality (Shapiro–Wilk test) and homogeneity of variances (F-test), an unpaired *t*-test was used for data meeting both assumptions; otherwise, the Mann–Whitney U test was employed. *p* ≤ 0.05 was considered a significant difference. 0.05 < *p* < 0.1 denoted a statistical trend.

## 3. Results

### 3.1. Goat Milk Formula Powder Improves the Growth Performance of Neonatal Piglets

As summarized in Table 3, compared with the CON group, the GMF group exhibited significantly greater final body weight (*p* < 0.05), with a trend toward improvement in total weight gain, ADG, ADFI, and feed conversion ratio (FCR) (0.05 < *p* < 0.1).

### 3.2. Goat Milk Formula Powder Influences Serum Biochemical Indexes of Neonatal Piglets

The impact of goat milk formula powder on piglet serum biochemical profiles is presented in Table 4. The GMF group had the trend to reduce ALT content (0.05 < *p* < 0.1), while significantly elevating CRP4 concentrations (*p* < 0.05). Immune profiling demonstrated that GMF group increased serum IgA (*p* < 0.01) and markedly elevated IgG and IgM levels (*p* < 0.001).

### 3.3. Goat Milk Formula Powder Improves Amino Acid Metabolism in Neonatal Piglets

**Figure 1:**
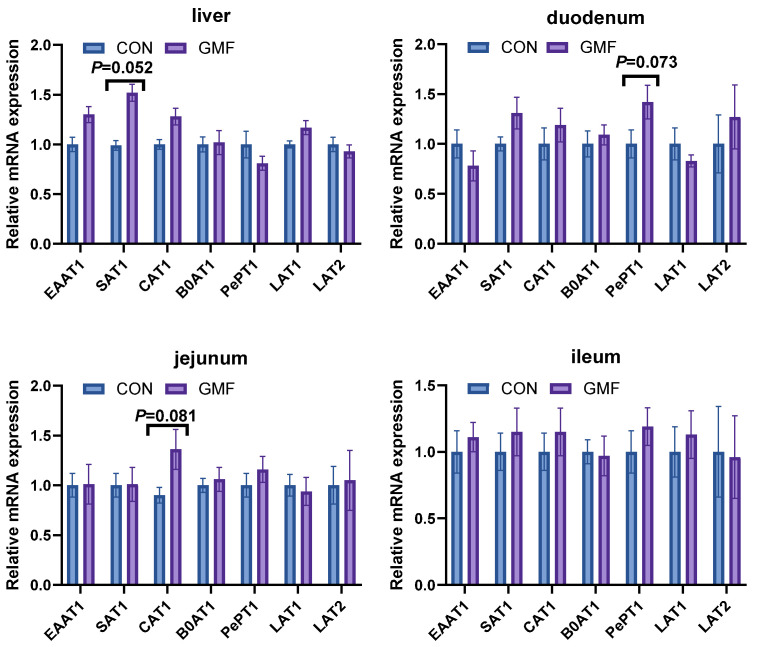
Effect of different formula milk powder on liver and intestine mRNA expression of amino acid transporters. CON, control group, standard formula milk powder; GMF, goat milk formula powder. *EAAT1*, excitatory amino acid transporter 1; *SAT1*, spermine N1-acetyltransferase 1; *CAT1*; cationic amino acid transporter 1; *B0AT1*, neutral amino acid transporter; *PePT1*, peptide transporter 1; *LAT1*, L-type amino acid transporter 1; *LAT2*, L-type amino acid transporter. Results are all expressed as means ± SEM (n = 8), and 0.05 < *p* < 0.1 was considered to have an increasing trend.

According to the data in Table 5, compared with the CON group, the GMF group significantly altered serum amino acid profiles, increasing essential amino acids, Thr, Glu, Ala, Val, Cys, Ile, Lys, and total amino acids levels (*p* < 0.05), while decreasing Tau and Tyr (*p* < 0.05), Leu, Asp, and Pro levels (0.05 < *p* < 0.1). To further investigate the underlying mechanisms, gene expression analysis was performed. As illustrated in Figure 1, gene expression analysis indicated upward trends in mRNA expression of hepatic *SAT1*, duodenal *PePT1*, and jejunum *CAT1* (0.05 < *p* < 0.1).

### 3.4. Goat Milk Formula Powder Modulates Serum Long-Chain Fatty Acids in Neonatal Piglets

Fatty acid composition results are listed in Table 6. Compared with the CON group, the GMF group significantly reduced the contents of C12:0, C16:0, C18:1n9c and saturated fatty acids (*p* < 0.05), while significantly increasing C20:4n6 and C22:6n3 (*p* < 0.05), with a trend toward higher contents of C20:2 and C20:0 (0.05 < *p* < 0.1).

### 3.5. Goat Milk Formula Powder Enhances the Contents of Intestinal Digestive Enzymes in Neonatal Piglets

As shown in Table 7, compared with the CON group, the trypsin content was significantly elevated in the GMF group (*p* < 0.05), while no significant differences were observed in the contents of α-amylase, chymotrypsin, lactase, or lipase (*p* > 0.05).

### 3.6. Goat Milk Formula Powder Regulates Intestinal Microbiota in Neonatal Piglets

**Figure 2:**
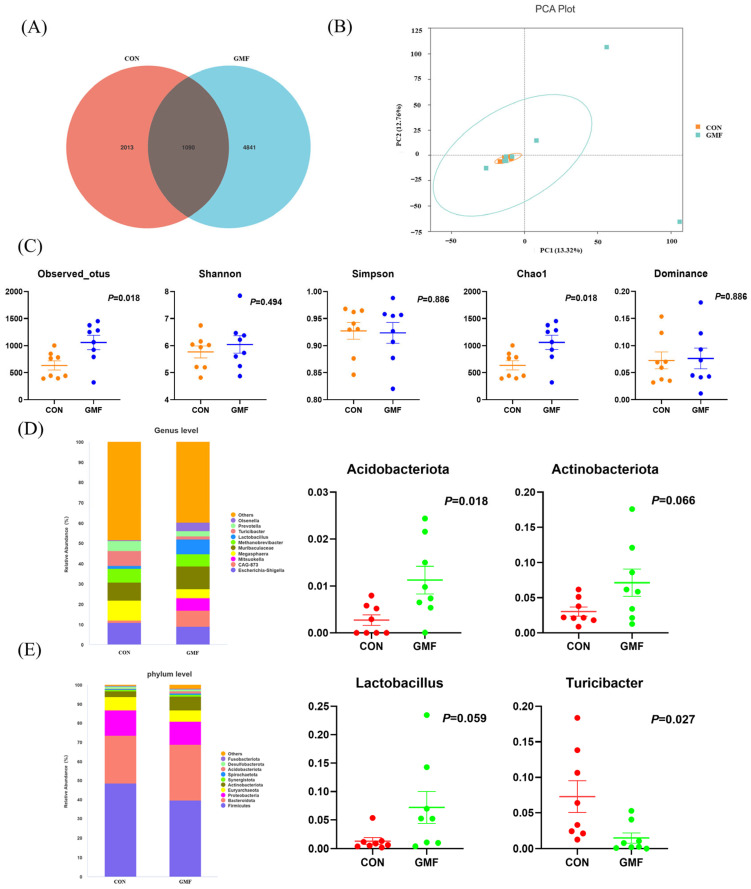
Goat milk powder can alter the relative abundance of the colonic microbial flora in piglets. (**A**) Venn diagram showing unique and shared OTUs (Operational Taxonomic Units) of gut microbiota in piglets; (**B**) Principal Component Analysis (PCA) of colonic Microbiota Beta Diversity; (**C**) Alpha-diversity analysis plots (*Chao 1*, *Dominance*, *Observed_features*, *Pielou_e*, *Shannon*, *Simpson*); (**D**) Relative abundance of the top 10 phyla in each dietary group (top) and relative abundance of significant microbial groups (bottom); (**E**) Relative abundance of the top 10 genera in each dietary group (top) and relative abundance of significant microbial groups (bottom). CON, control group, standard formula milk powder; GMF, goat milk formula powder. Results are all expressed as means ± SEM (n = 8), and *p* ≤ 0.05 was considered to be a significant difference.

Between the CON and GMF groups, gut microbiota profiling (Figure 2A) identified 1090 shared operational taxonomic units (OTUs), with 2013 and 4841 unique OTUs, respectively. Principal Coordinate Analysis (PCA, Figure 2B) showed that the β-diversity of the CON group overlapped with that of the GMF group. Alpha diversity indices (Chao1 and observed features) were significantly greater than CON group (*p* < 0.05, Figure 2C). At the phylum level (Figure 2D), GMF group elevated the relative abundance of *Acidobacteriota* (*p* < 0.05) and showed trends toward greater *Actinobacterota* (0.05 < *p* < 0.1). Genus-level analysis (Figure 2E) showed reduced *Turicibacter* (*p* < 0.05) and trends toward elevated the relative abundance of *Lactobacillus* and *Olsenella* (0.05 < *p* < 0.1).

**Figure 3:**
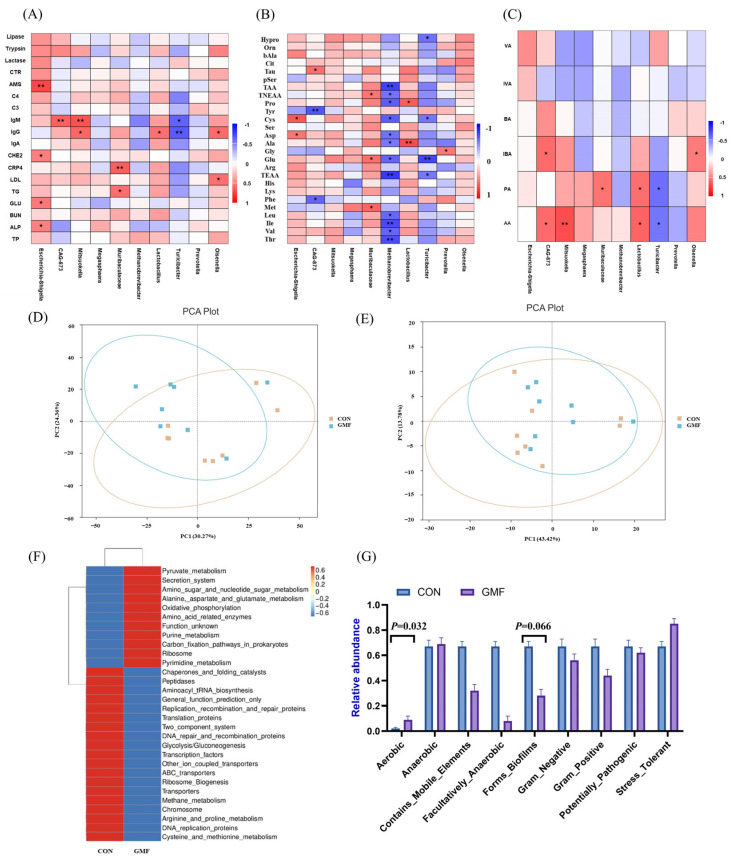
G Heatmap of Spearman’s correlations analysis and analysis of metabolic functions and phenotypes of colonic microbiota. Correlation analysis between colonic microbiota (**A**) serum biochemical indices, immune markers, and digestive enzyme activity; (**B**) circulating free amino acids; (**C**) short-chain fatty acid profiles at the genus level; (**D**,**E**) The PCA based on EC and pathways of PICRUSt2; (**F**) Clustering analysis of the relative abundance of functional pathways based on KEGG (Kyoto Encyclopedia of Genes and Genomes) annotation predicted by PICRUSt2; (**G**) Metabolic phenotype analysis using BugBase. TAA, Total amino acids; TNEAA, Total non-essential amino acids; TEAA, Total essential amino acids; AA, Acetic acid; PA, Propionic acid; IBA, Isobutyric acid; BA, Butyric acid; IVA, Isovaleric acid; VA, Valeric acid.; CON, control group, standard formula milk powder; GMF, goat milk formula powder, * *p* < 0.05 and ** *p* < 0.01. Results are all expressed as means ± SEM (n = 8), and *p* ≤ 0.05 was considered to be a significant difference.

Heatmap of Spearman’s correlation analysis is illustrated in Figure 3A–C. *Olsenella* abundance shows a positive association with serum LDL, IgG and IBA levels (*p* < 0.05). *Prevotella* abundance is positively correlated with Gly (*p* < 0.05). *Turicibacter* abundance is negatively correlated with serum IgG (*p* < 0.01) and IgM (*p* < 0.05), total essential amino acids (TEAA) (*p* < 0.05), Cys (*p* < 0.05), and Glu (*p* < 0.01), acetic acid (AA) and propionic acid (PA) (*p* < 0.05). *Lactobacillus* showed positive association with IgG (*p* < 0.05), Ala (*p* < 0.01) and Pro (*p* < 0.05), AA and PA (*p* < 0.05). *Methanobrevibacter* abundance is negatively correlated with Thr, Ile, TEAA and total amino acids (TAA) (*p* < 0.01), Val, Leu, Glu, Ala, Asp, Cys, Pro and total non-essential amino acids (TNEAA) (*p* < 0.05). *Muribaculaceae* abundance shows a positive association with CRP4 (*p* < 0.01) and TG (*p* < 0.05), Met, Glu, TNEAA, and PA (*p* < 0.05). CAG-873 abundance is negatively correlated with Phe (*p* < 0.05) and Thr (*p* < 0.01), and CAG-873 abundance shows a positive association with Tau (*p* < 0.05). *Mitsuokella* is positively correlated with serum IgG (*p* < 0.05), IgM (*p* < 0.01), and AA (*p* < 0.01). CAG-873 exhibited strong associations with IgM (*p* < 0.01) and *CAG-873* abundance shows a positive association with Isobutyric acid (IBA) and AA (*p* < 0.05). *Escherichia-Shigella* abundance correlated with multiple biomarkers, including AMS (*p* < 0.01) and ALP, GLU, CHE2 (*p* < 0.05), and *Escherichia-Shigella* shows a positive association with Asp and Cys (*p* < 0.05).

Based on the PICRUSt2 analysis of EC numbers and pathways, the Principal Coordinate Analysis (PCA, Figure 3D,E) plot showed an overlap between the CON and GMF groups. Functional analysis revealed KEGG pathway divergence between groups (Figure 3F). GMF group enhanced aerobic bacterial richness (Figure 3G) and a trend toward biofilm-forming capacity (0.05 < *p* < 0.1).

### 3.7. Goat Milk Formula Powder Increases the Production of Short-Chain Fatty Acids in Neonatal Piglet

As presented in Table 8, short-chain fatty acid (SCFA) analysis revealed elevated acetic acid content and propionic acid contents in GMF group (*p* < 0.05) with no significant changes in isobutyric acid, butyric acid, isovaleric acid and valeric acid contents (*p* > 0.05).

## 4. Discussion

Growth performance directly reflects the health status of piglets. Studies confirm that GMF supports infant growth and safety [24]. Roy et al. [25] compared gastric emptying rates of cow milk, goat milk and sheep milk, revealing faster clearance for goat milk forms gastric curds that modulate nutrient release. Goat milk curds release proteins and lipids more efficiently than cow milk, enhancing small intestine absorption. In this study, GMF group piglets exhibited marginally greater weight gain and feed intake than CON group, likely linked to improved nutrient digestion. Maximino et al. [26] demonstrated that GMF was well-tolerated and safe in human infants, supporting adequate growth with a low incidence of gastrointestinal symptoms. Serum biomarkers reflect systemic health and organ function [27]. Colostrum provides passive immunity to neonatal piglets [28], with 70–80% of immunoglobulins absorbed into circulation [29,30]. The GMF group piglets showed elevated IgA, IgG, IgM, and CRP4 levels, indicating immune enhancement. The results of Xu et al. [15] were consistent with those of the present study. van Lee et al. [31] found that GMF, similar to human milk, supported longer daytime sleep duration compared to CMF, supporting its use as a valuable alternative when human milk is unavailable. Rutherfurd et al. [32] reported that GMF promoted better absorption of certain minerals than CMF in 3-week-old piglets.

Serum free amino acids reflect nutritional status and are influenced by intestinal absorption, cellular metabolism, and transmembrane transport activity [33]. Amino acid transporters (AATs) mediate uptake and regulate energy balance, protein synthesis and redox [34,35]. Notably, peptide transporter *PEPT1* facilitates di/tripeptide absorption [21]. Circulating amino acids serve dual roles as protein precursors and immune modulators [36,37]. Hodgkinson et al. [38] found distinct sets of peptides between cow milk and goat milk, which may explain the differences in their digestibility. Maathuiset et al. [39] reported slower initial protein digestion in GMP under infant conditions; elevated amino acid levels suggest enhanced metabolism, promoting protein deposition, immune function, and energy utilization. Lipid metabolism encompasses the synthesis and degradation of fatty acids, triglycerides, and cholesterol [40]. Free fatty acids (FFAs) are derived primarily from dietary sources and serve as key energy substrates [41]. Saturated fatty acids (SFAs) stabilize cell membranes against oxidation [42], while polyunsaturated fatty acids (PUFAs, e.g., C20:4n6 and C22:6n3) are essential for membrane integrity [43]. Goat milk contains abundant phospholipids, including hexosylceramide, dihexosylceramide, sphingomyelin, ceramide, and phosphatidylcholine [44]. Wang et al. [45] demonstrated that GMF diets elevate serum triglyceride levels and modulate lipid metabolism-related gene expression in piglets, aligning with our findings. Protein digestion relies on enzymatic hydrolysis. Digestive enzymes, synthesized as inactive zymogens (e.g., trypsinogen), are activated in the intestine to cleave proteins into absorbable peptides [46,47,48]. In this study, GMF feeding significantly increased intestinal trypsin levels, which enhanced proteolytic efficiency and accelerated the cleavage of goat milk proteins into small-molecule peptides and free amino acids. These degradation products were efficiently absorbed through *PePT1* and amino acid transporters (AATs) in the small intestinal epithelial cells, ultimately leading to elevated levels of certain free amino acids in the serum. This process improved amino acid utilization in piglets; without increasing total serum protein levels, it provided sufficient precursors for immune cell synthesis, thereby mediating the increase in immune indicators such as IgA, IgG and IgM in the GMF group piglets.

Gut microbiota analysis identified 5764 OTUs, with 2013 and 4841 unique to the CON and the GMF groups, respectively. Greater Chao1 diversity of GMF piglets indicated greater microbial richness—a hallmark of gut health linked to reduced inflammation and metabolic dysfunction [49]. The gut microbiota critically regulates host glucose and lipid metabolism [40]. *Acidobacteriota*, which is capable of degrading plant-derived chitin, xylan, cellulose, and hemicellulose while participating in sulfur cycling [50]. *Actinobacteriota* (Gram-positive bacteria), which exhibit filamentous morphology and possess exceptionally high genomic guanine plus cytosine (G+C) content, play a crucial role in maintaining intestinal homeostasis [51,52]. Wang et al. [53] demonstrated that goat milk exerts a more profound influence on gut microbiota than cow milk, with *Akkermansia* showing significant enrichment exclusively in the GMF group—a phenomenon potentially attributable to the distinctive functional properties of goat milk, using murine models. Comparative analysis revealed that, relative to breastfed cubs, Siberian tiger cubs fed with goat milk exhibited distinct gut microbial metabolic shifts—reduced carbohydrate metabolism, translation, and DNA replication processes—concomitant with enhanced amino acid metabolism, membrane transport, as well as cofactor and vitamin metabolism [54]. *Turicibacter* (*Firmicutes phylum*, *Erysipelotrichaceae* family) modulates host bile acid and lipid metabolism [55,56]. GMF enriched beneficial genera (e.g., *Blautia*, *Roseburia*, and *Muribaculum*), mirroring patterns observed in breast milk-fed infants [57]. Clinical trials in Brazil and Mexico demonstrated that GMF reduces gastrointestinal symptoms, likely mediated by gut microbiota modulation [58]. *Lactobacillus*, a prominent probiotic, regulates host immunity and gut balance via bacteriocin secretion and acid environment maintenance [53,54,55,59,60,61]. GMF feeding promotes *Lactobacillus* proliferation while suppressing pathogens, as shown in murine models [62]. *Olsenella* (*Atopobiaceae* family) correlates with acetate levels, suggesting metabolic synergy with *Lactobacillus* [63,64].

SCFAs, produced by microbial fiber fermentation, enhance energy metabolism, intestinal health and immune function [65]. As end products of microbial breakdown of dietary fibers in the gut, SCFAs can be absorbed by the intestines and utilized by host cells as energy sources. Propionate supports gluconeogenesis and mitigates obesity [66], while butyrate fuels colonocyte integrity and suppresses inflammation [67]. The results indicated that acetate and propionate levels in the feces of piglets in the GMF group were significantly increased, enhancing the body’s anti-inflammatory capacity, maintenance of intestinal epithelial barrier function and energy stability. This study demonstrates that GMF modulates the gut microbiota in piglets, promoting the production of SCFAs, specifically acetate and propionate acid. These SCFAs potentially enhance immune-related gene expression and regulate energy metabolism, respectively, collectively mediating the observed improvements in immune markers (CRP4, IgA, IgG, IgM) and lipid metabolism. A key limitation of this study is its underpowered nature due to the constrained sample size and the trial duration. Therefore, these findings should be interpreted with caution. In the future, we will increase the sample size and extend the study period in subsequent trials to further investigate the underlying mechanisms of GMF on immunity.

## 5. Conclusions

This study demonstrates that goat milk formula shows a promising enhancement in the growth performance of neonatal piglets by improving immunity, optimizing amino acid and fatty acid metabolism and modulating intestinal microbiota, demonstrating goat milk formula superiority over cow milk formula.

## Figures and Tables

**Table 1:** Nutritional component values of different infant formula milk powders.

Items	CON	GMF
Energy, KJ	2077	2132
Protein, g	12.0	10.5
Fat, g	24.9	28
Linoleic Acid, g	1.87	4
Alpha-Linolenic Acid, mg	291	400
Carbohydrates, g	56.0	52.2
Vitamins		
Vitamin A, μg RE	374	385
Vitamin D, μg	6.85	12.9
Vitamin E, mg α-TE	3.32	6
Vitamin K_1_, μg	29.1	55
Vitamin B_1_, μg	374	550
Vitamin B_2_, μg	498	650
Vitamin B_6_, μg	218	380
Vitamin B_12_, μg	0.644	1.5
Niacin	-	3500
Folate, μg	66.5	78
Pantothenic Acid, μg	2492	2618
Vitamin C, mg	60.2	64
Biotin, μg	10.4	10
Minerals		
Sodium, mg	125	187
Potassium, mg	322	459
Copper, μg	228.5	381
Magnesium, mg	31.2	32
Iron, mg	2.70	4.7
Zinc, mg	3.12	3.3
Manganese, μg	31.2	32.3
Calcium, mg	270	350
Total phosphorus, mg	145	220
Iodine, μg	66.5	100
Chlorine, mg	312	322
Selenium, μg	12.46	19.5
Optional Components		
Choline, mg	45.7	-
Creatine, mg	29.1	-
L-Carnitine, mg	10.4	11
Docosahexaenoic Acid, % of Total Fatty Acids	0.30	-
DHA, mg	-	96
Arachidonic Acid, % of Total Fatty Acids	0.35	-
Inositol, mg	-	45
Taurine, mg	-	30
ARA/AA, mg	-	120
Galacto-oligosaccharides, g	-	1.82
Fructo-oligosaccharides, g	-	1.94
OPO, g	-	6
Nucleotides, mg	25.0	35

CON, control group, standard formula milk powder; GMF, goat milk formula powder. “-” represents “no data”. DHA, Docosahexaenoic Acid; ARA/AA, Arachidonic Acid; OPO, 1,3-Dioleoyl-2-Palmitoyl Triglyceride. The above values represent the nutritional content per 100 g of formula powder. In the prepared state, every 100 mL contains 13.5 g of infant formula powder (CON).

**Table 2:** Gene and primer sequences.

Genes	Primer Sequences, 5′-3′	Size (bp)	TA (°C)	Accession Number
*EAAT1*	F: GATGGGACCGCCCTCTAT	105	58	NM_001289944.1
	R: CGTGGCTGTGATGCTGATG			
*SAT1*	F: AACAGTCTCCAACCCTCTTCAC	166	58	NM_214358.1
	R: GCTTTGGCATAGGATCAGAAAT			
*CAT1*	F: GCCTGAACAATGCCACGAAG	109	60	NM_001012613.1
	R: CCCACGAAGGCATAGAAGCA			
*B* *0* *AT1*	F: AAGGCCCAGTACATGCTCAC	102	60	XM_003359855.4
	R: CATAAATGCCCCTCCACCGT			
*PePT1*	F: CAGACTTCGACCACAACGGA	99	60	NM_214347.1
	R: TTATCCCGCCAGTACCCAGA			
*LAT1*	F: GAGCAGGTGAAGCTGAAGAAGG	174	60	NM_001110421.1
	R: CCCAAAGACGGAGAAGAGGC			
*LAT2*	F: ACAGGAGTGCCCGTCTATT	90	58	XM_021099240.1
	R: GCTCACCAGGGTCAACAAC			

*EAAT1*, excitatory amino acid transporter 1; *SAT1*, spermine N1-acetyltransferase 1; *CAT1*; cationic amino acid transporter 1; *B0AT1*, neutral amino acid transporter B0AT1; *PePT1*, peptide transporter 1; *LAT1*, L-type amino acid transporter 1; *LAT2*, L-type amino acid transporter.

**Table 3:** Effect of different formula milk powder on growth performance of piglets.

Items	CON	GMF	*p*-Value
Initial weight, g	2189.4 ± 44.3	2255 ± 45.98	0.321
Final weight, g	2926.3 ± 111	3255 ± 77.02	0.029
Total weight gain, g	736.9 ± 85	1000 ± 93.3	0.056
ADG, g/d	52.63 ± 6.07	71.43 ± 6.66	0.056
ADFI, g/d	78.23 ± 5.05	92.09 ± 4.42	0.058
FCR	1.56 ± 0.10	1.33 ± 0.06	0.064

CON, control group, standard formula milk powder; GMF, goat milk formula powder. ADG, average daily gain; ADFI, average daily feed intake; FCR, feed conversion ratio. Results are all expressed as means ± SEM (n = 8), and *p* ≤ 0.05 was considered to be a significant difference.

**Table 4:** Effect of different formula milk powder on the serum biochemical indexes of piglets.

Items	CON	GMF	*p*-Value
TP, g/L	48.17 ± 2.00	48.78 ± 2.97	0.869
BUN, mmol/L	0.95 ± 0.15	0.95 ± 0.15	1.000
ALB, g/L	23.83 ± 1.62	22.76 ± 0.95	0.581
ALT, U/L	67.38 ± 6.76	53.99 ± 3.28	0.097
GLU, mmol/L	6.84 ± 0.35	7.15 ± 0.42	0.573
AST, U/L	162.13 ± 23.98	133.63 ± 19.84	0.375
ALP, U/L	444.25 ± 47.92	383.50 ± 33.00	0.314
CHOL, mmol/L	2.83 ± 0.20	2.76 ± 0.15	0.784
CHE2, U/L	473.63 ± 25.88	507.00 ± 29.05	0.405
LDL-C, mmol/L	1.29 ± 0.13	1.48 ± 0.10	0.239
TG, mmol/L	0.59 ± 0.10	0.76 ± 0.11	0.257
LIPC, U/L	10.01 ± 2.09	17.95 ± 4.89	0.158
HDL-C, mmol/L	1.69 ± 0.10	1.50 ± 0.08	0.165
CRP4, mg/L	0.12 ± 0.02	0.18 ± 0.02	0.041
IgA, μg/mL	305.14 ± 34.14	489.66 ± 44.52	0.005
IgG, mg/mL	9.87 ± 0.64	16.03 ± 0.83	<0.001
IgM, mg/mL	9.60 ± 0.37	13.82 ± 0.74	<0.001
C3, g/L	0.02 ± 00.00	0.02 ± 0.00	0.781
C4, g/L	0.03 ± 0.00	0.04 ± 0.00	0.166

CON, control group, standard formula milk powder; GMF, goat milk formula powder. TP, total protein; BUN, urea; ALB, albumin; ALT, alanine aminotransferase; GLU, glucose; AST, aspartate aminotransferase; ALP, alkaline phosphatase; CHOL, total cholesterol; CHE2, cholinesterase; LDL-C, low-density lipoprotein; TG, triglyceride; LIPC, Hepatic Lipase; HDL-C, high-density lipoprotein; CRP4, C-reactive protein; IgA, immunoglobulin A; IgG, immunoglobulin G; IgM, immunoglobulin M; C3, Complement C3; C4, Complement C4. Results are all expressed as means ± SEM (n = 8), and *p* ≤ 0.05 was considered to be a significant difference.

**Table 5:** Effect of different formula milk powder on serum free amino acids in piglets.

Items, μg/mL	CON	GMF	*p*-Value
Essential amino acids			
Thr	0.044 ± 0.008	0.070 ± 0.007	0.025
Val	0.017 ± 0.001	0.025 ± 0.002	0.011
Met	0.010 ± 0.001	0.013 ± 0.001	0.046
Phe			
Leu	0.014 ± 0.001	0.018 ± 0.002	0.099
Met	0.008 ± 0.000	0.009 ± 0.001	0.447
Phe	0.008 ± 0.001	0.007 ± 0.002	0.392
Lys	0.021 ± 0.002	0.028 ± 0.002	0.028
His	0.010 ± 0.001	0.012 ± 0.001	0.116
Arg	0.009 ± 0.001	0.009 ± 0.001	0.718
Non-essential amino acids			
Glu	0.024 ± 0.002	0.031 ± 0.002	0.040
Gly	0.111 ± 0.007	0.108 ± 0.009	0.823
Ala	0.053 ± 0.004	0.074 ± 0.009	0.049
Asp	0.004 ± 0.001	0.007 ± 0.001	0.072
Pro	0.027 ± 0.001	0.033 ± 0.003	0.071
Cys	0.004 ± 0.001	0.007 ± 0.001	0.024
Ser	0.026 ± 0.001	0.029 ± 0.002	0.314
Tyr	0.008 ± 0.001	0.006 ± 0.001	0.040
Total essential amino acids	0.140 ± 0.010	0.190 ± 0.015	0.018
Total non-essential amino acids	0.257 ± 0.009	0.295 ± 0.023	0.140
Total amino acids	0.397 ± 0.019	0.485 ± 0.035	0.044
Amino acid derivatives and metabolites			
p-Ser	0.003 ± 0.000	0.004 ± 0.001	0.265
Cit	0.019 ± 0.001	0.020 ± 0.002	0.565
b-Ala	0.002 ± 0.000	0.002 ± 0.000	0.542
Orn	0.006 ± 0.000	0.006 ± 0.001	0.431
Tau	0.125 ± 0.002	0.026 ± 0.003	0.003
Hypro	0.014 ± 0.002	0.017 ± 0.001	0.230

CON, control group, standard formula milk powder; GMF, goat milk formula powder. Thr, Threonine; Val, Valine; Ile, Isoleucine; Leu, Leucine; Met, Methionine; Phe, Phenylalanine; Lys, Lysine; His, Histidine; Arg, Arginine; Glu, Glutamic; Gly, Glycine; Ala, Alanine; Asp, Aspartic acid; Pro, Proline; Cys, Cysteine; Ser, Serine; Tyr, Tyrosine; p-Ser, Phosphoserine; Cit, Citrulline; b-Ala, Beta-Alanine; Orn, Ornithine; Tau, Taurine; Hypro, Hydroxyproline. Results are all expressed as means ± SEM (n = 8), and *p* ≤ 0.05 was considered to be a significant difference.

**Table 6:** Effects of different formula milk powder on serum long-chain fatty acids in piglets.

Items, %	CON	GMF	*p*-Value
C12:0	0.64 ± 0.12	0.31 ± 0.00	0.031
C14:0	0.88 ± 0.13	0.72 ± 0.07	0.282
C16:0	24.47 ± 0.43	22.86 ± 0.40	0.016
C16:1	0.39 ± 0.03	0.36 ± 0.03	0.471
C17:0	0.34 ± 0.00	0.36 ± 0.01	0.198
C18:0	14.96 ± 0.50	15.09 ± 0.51	0.854
C18:1n9t	0.17 ± 0.01	0.17 ± 0.01	0.949
C18:1n9c	20.04 ± 0.58	17.69 ± 0.46	0.007
C18:2n6c	20.74 ± 0.34	21.23 ± 0.28	0.281
C20:0	0.17 ± 0.01	0.15 ± 0.01	0.076
C20:1	0.17 ± 0.01	0.16 ± 0.01	0.556
C18:3n3	0.93 ± 0.05	0.88 ± 0.05	0.510
C20:2	0.29 ± 0.02	0.35 ± 0.02	0.078
C20:3n6	0.52 ± 0.04	0.56 ± 0.04	0.563
C20:4n6	10.74 ± 0.41	12.42 ± 0.35	0.008
C22:6n3	4.63 ± 0.16	5.92 ± 0.32	0.003
SFA	41.38 ± 0.30	39.27 ± 0.36	<0.001
MUFA	58.43 ± 0.27	59.53 ± 0.44	0.053

CON, control group, standard formula milk powder; GMF, goat milk formula powder. SFA (saturated fatty acid) = C12:0 + C14:0 + C16:0 + C17:0 + C18:0 + C20:0; MUFA (monounsaturated fatty acid) = C16:1 + C18:1n9c + C18:1n9t + C18:2n6c + C18:3n3 + C20:3n6 + C20:4n6 + C22:6n3. Results are all expressed as means ± SEM (n = 8), and *p* ≤ 0.05 was considered to be a significant difference.

**Table 7:** Effect of different formula milk powder on intestinal digestive enzymes in piglets.

Items	CON	GMF	*p*-Value
α-Amylase, ng/g prot	898.59 ± 38.65	921.97 ± 28.25	0.633
Chymotrypsin, ng/g prot	203.96 ± 7.19	210.90 ± 10.25	0.588
Lactase, pg/mg prot	148.20 ± 7.10	138.21 ± 6.01	0.301
Trypsin, ng/mg prot	3.48 ± 0.09	3.99 ± 0.14	0.009
Lipase, ng/g prot	92.83 ± 4.26	98.96 ± 3.62	0.291

CON, control group, standard formula milk powder; GMF, goat milk formula powder. Results are all expressed as means ± SEM (n = 8), and *p* ≤ 0.05 was considered to be a significant difference.

**Table 8:** Effect of different formula milk powder on short-chain fatty acids in piglet feces.

Items, μg/g	CON	GMF	*p*-Value
Acetic acid	1755.98 ± 240.08	2644.87 ± 116.91	0.007
Propionic acid	3.48 ± 0.57	5.42 ± 0.59	0.035
Isobutyric acid	1.20 ± 0.21	0.98 ± 0.17	0.546
Butyric acid	59.13 ± 15.00	86.69 ± 15.73	0.411
Isovaleric acid	2.84 ± 0.56	3.22 ± 0.72	0.696
Valeric acid	20.44 ± 5.63	21.74 ± 5.07	0.867

CON, control group, standard formula milk powder; GMF, goat milk formula powder. Results are all expressed as means ± SEM (n = 8), and *p* ≤ 0.05 was considered to be a significant difference.

## Data Availability

The repository/repository names and accession number (s) are available in the NCBI SRA database under the identifier PRJNA1255375.

## Abbreviations

CON	standard formula milk powder
GMF	goat milk formula powder
ADG	average daily gain
ADFI	average daily feed intake
FCR	feed conversion ratio
TP	total protein
BUN	urea
ALB	albumin
ALT	alanine aminotransferase
GLU	glucose
AST	aspartate aminotransferase
ALP	alkaline phosphatase
CRP4	C-reactive protein
CHOL	total cholesterol
CHE2	cholinesterase
LDL-C	low-density lipoprotein
TG	triglycerides
LIPC	hepatic lipase
IgA	immunoglobulin A
IgG	immunoglobulin G
IgM	immunoglobulin M
C3	complement C3
C4	complement C4
SFA	saturated fatty acid
TAA	total amino acids
TEAA	total essential amino acid
TNEAA	total non-essential amino acid
AA	acetic acid
PA	propionic acid
IBA	isobutyric acid
BA	butyric acid
IVA	isovaleric acid
VA	valeric acid
SCFA	short-chain fatty acid
*EAAT1*	excitatory amino acid transporter 1
*SAT1*	spermine N1-acetyltransferase 1
*CAT1*	cationic amino acid transporter 1
*B0AT1*	neutral amino acid transporter B0AT1
*PePT1*,	peptide transporter 1
*LAT1*	L-type amino acid transporter 1
*LAT2*	L-type amino acid transporter 2
G+C	guanine plus cytosine
AATs	amino acid transporters
